# Associations between pre-to-post TMS-evoked potential changes and motor sequence learning across retention intervals

**DOI:** 10.3389/fnhum.2026.1925178

**Published:** 2026-09-23

**Authors:** Fransina Christina King, Narayanankutty Naduvilidam, Milos R. Ljubisavljevic, Fatima Yousif Ismail

**Affiliations:** 1 Department of Physiology, College of Medicine and Health Sciences (CMHS), United Arab Emirates University, Al Ain, United Arab Emirates; 2 Department of Pediatrics, College of Medicine and Health Sciences (CMHS), United Arab Emirates University, Al Ain, United Arab Emirates; 3 Department of Neurology (Adjunct), Johns Hopkins University School of Medicine, Baltimore, MD, United States

**Keywords:** electroencephalography, motor learning, motor sequence learning, non-dominant hand, retention, TMS-evoked potential, transcranial magnetic stimulation

## Abstract

Motor sequence learning involves practice-related changes in behavior and neural activity, yet the neurophysiological markers associated with later retention remain unclear. TMS-evoked potentials (TEPs) provide a measure of cortical responses to stimulation and may help characterize physiological-behavioral relationships across different learning timescales. Whether these associations differ across retention intervals remains unclear. We examined whether baseline TMS-EEG measures or task-related changes in right primary motor cortex (M1) TEPs are associated with initial acquisition and subsequent performance change across short and long-term retention intervals during a non-dominant hand motor sequence task. Seventeen healthy right-handed adults performed a five-element finger-tapping sequence with the left hand across three sessions: Day 1 (acquisition), Day 2 (short-term retention), and Day 7 (long-term retention). Single-pulse TMS-EEG was acquired before and after task performance over right M1 at each session. Four TEP components (N45, N100, P180, and N280) were analyzed. Day 1 training was associated with increases in PR and ACC, together with improved accuracy-adjusted efficiency, and slope dynamics remained broadly stable across later test sessions. Acute pre-to-post right-M1 TEP changes were not associated with rapid within-session acquisition. Pre-to-post P180 TEP change showed a nominal association with subsequent efficiency change, with a stronger nominal pattern over the 24-h interval than over the 5-day interval. However, neither the P180 dTEP effect nor the Interval × dTEP interaction survived FDR correction, and both were sensitive to participant-level influence and bootstrap resampling. These findings suggest that right-M1 TEP measures showed different relationships with rapid within-session acquisition and between-session performance change. Late P180 changes may be related to performance change across different retention intervals, but whether they reflect time-dependent consolidation processes remains to be established.

## 1 Introduction

Motor skill learning, the process by which repetitive practice leads to faster and more accurate movement, is fundamental to everyday function and underpins many rehabilitation strategies. Sequential motor learning, in which individuals acquire specific movement orders through practice, follows a two-phase trajectory. During an initial fast-learning phase, performance improves rapidly as learners adjust movements to sensory feedback, correct errors, and begin to bind successive responses into more efficient action units or chunks (Doyon and Benali, 2005; Hikosaka et al., 1999; Karni et al., 1998). With continued practice, a slower phase emerges in which gains become more incremental and may reflect ongoing stabilization and consolidation of motor memory (Reis et al., 2009; Doyon et al., 2009; Walker et al., 2002).

Understanding the neural substrates of these phases is important because individual differences in learning capacity influence recovery after injury. Post-injury motor rehabilitation relies on the same plasticity mechanisms that support skill acquisition in healthy individuals, and identifying markers that distinguish learners who consolidate gains from those who do not could inform the timing and evaluation of therapeutic interventions (Maier et al., 2019).

Neurophysiological and neuroimaging studies identify the primary motor cortex (M1) as an important site of plasticity during motor skill acquisition and consolidation. M1 controls muscle activity through its corticospinal projections, and its activity and organization change with the learning and maintenance of sequential movements (Karni et al., 1998; Floyer-Lea and Matthews, 2004; Dayan and Cohen, 2011). Yet motor sequence learning engages distributed networks including premotor, parietal, striatal, cerebellar, and hippocampal regions, with contributions differing across early and later learning stages (Penhune and Steele, 2012; Albouy et al., 2013; Hikosaka et al., 2002). Clarifying how local cortical excitability and network dynamics change across learning is important for translational neurophysiology.

Transcranial magnetic stimulation combined with electroencephalography (TMS-EEG) probes these dynamics directly. A focal TMS pulse perturbs cortical tissue and evokes time-locked EEG responses known as TMS-evoked potentials (TEPs), which reflect the functional state of the stimulated cortex and, through the propagation of activity to connected regions, network-level dynamics (Komssi and Kähkönen, 2006; Rogasch and Fitzgerald, 2013; Ilmoniemi and Kičić, 2010). TEPs exhibit characteristic morphology with negative and positive peaks at specific latencies, including the N45, N100, P180, and N280. Early components such as N45 and N100 have been linked to inhibitory cortical processes, although these associations remain methodologically constrained, whereas later components are interpreted as reflecting broader network interactions (Premoli et al., 2014; Premoli et al., 2017; Vernet et al., 2013; Tremblay et al., 2019). When applied over M1, TMS-EEG can provide millisecond-resolved responses that may reflect local and network-level activity, and may capture training-related changes relevant to motor learning.

Despite increased use of TMS-EEG to study motor learning, several gaps remain. Existing work has primarily examined changes within a single session, and TEP dynamics across multiple sessions of motor learning remain less well characterized. Prior work has shown that TEP components in secondary motor cortex change with practice during sequence learning (Thong et al., 2025). Whether this association extends to the explicit motor sequence learning task examined here remains unclear. In addition, it is not known whether N100-behavior associations differ between short and long-term retention, although behavioral findings suggest that these retention-related processes may be dissociable (Doyon et al., 2009; Walker et al., 2002; Nishida and Walker, 2007). TMS-EEG studies of motor learning have also focused on the dominant hand. Consequently, less is known about TEP-behavioral relationships during learning with the non-dominant hand, where learning may engage broader neural networks and may provide greater dynamic range for detecting practice-related changes (Grafton et al., 2002; Pool et al., 2014). These considerations are particularly relevant when interpreting TEP changes across learning and retention, as interindividual variability and the state dependence of baseline TMS-EEG measures may influence the observed physiological-behavioral associations (Wang et al., 2026).

In this study, we combined single-pulse TMS-EEG with a sequential finger-tapping task performed with the non-dominant left hand. Across three sessions (Day 1 acquisition, Day 2 short-term retention, and Day 7 long-term retention), participants practiced a five-element sequence while TEPs were recorded from right M1 before and after task performance. Based on the evidence summarized above, our primary hypothesis was that pre-to-post changes in N100 amplitude would be associated with subsequent task-performance change and that the strength of this association would differ between the short and long-term retention intervals (Doyon et al., 2009; Walker et al., 2002). No directional prediction was specified regarding which interval would show the stronger association. We additionally examined two secondary questions concerning Day 1 acquisition and subsequent between-session performance. First, we tested whether baseline N100 amplitude was associated with Day 1 acquisition, consistent with the possibility that baseline TEP amplitude contributes to individual differences in initial learning. Second, we tested whether pre-to-post N100 changes measured on Day 1 were associated with subsequent between-session performance change. Such an association would be consistent with, but would not by itself establish, a role for acute TEP changes in processes contributing to performance change across shorter and longer intervals. N45, P180, and N280 were included as additional canonical TEP components to assess whether any observed associations extended beyond N100. Analyses involving these components were exploratory, and no component-specific directional predictions were made.

## 2 Materials and methods

### 2.1 Participants

Twenty-seven healthy right-handed adults were recruited and screened with the Edinburgh Handedness Inventory (Oldfield, 1971) and a standard TMS safety questionnaire (Rossi et al., 2021). Exclusion criteria were ambidexterity (*n* = 2), history of severe migraine (*n* = 2), prior head injury (*n* = 3), neurological or psychiatric disorders (*n* = 0), personal or family history of epilepsy (*n* = 0), other TMS contraindications (*n* = 0), or voluntary withdrawal (*n* = 3). The final study sample comprised 17 right-handed participants (laterality quotient > 80; mean age 34.3 ± 8.6 years; 6 female). All participants provided written informed consent. The study was approved by the institutional ethics committee (ERH-2020_6165 2020–19) in accordance with the Declaration of Helsinki.

### 2.2 Experimental setup

The sequential tapping task and stimulation triggers were controlled using E-Prime 3.0; event codes were sent via a Chronos interface to the TMS stimulator and the EEG acquisition system to ensure precise temporal alignment across all three systems. TMS was delivered with a MAG and More PMD70-pCool figure-of-eight coil connected to a MAG and More PowerMag stimulator (MAG and More GmbH, Munich, Germany). EEG was recorded using an ANT Neuro amplifier (EEGo MyLab, ANT Neuro, Hengelo, and The Netherlands).

### 2.3 Study design and procedure

Participants attended three sessions scheduled at the same time of day to minimize circadian influences. Session 1 (Day 1) represented the acquisition phase and included training and test blocks. Session 2 (Day 2), conducted approximately 24 h later, probed short-term retention. Session 3 (Day 7) assessed long-term interval retention-related performance dynamics. Each session began with a pre-task TMS-EEG assessment, followed by the motor task, and ended with an identical post-task TMS-EEG assessment. Resting motor threshold (RMT) was determined at the start of each session, and all subsequent stimulation intensities were scaled to that session-specific threshold.

### 2.4 Sequential motor learning task

Participants sat ~ 60 cm from a 24-inch monitor and placed the non-dominant left hand on four keys ('1′-'4′) of a standard QWERTY keyboard. The left little finger rested on “1,” the ring finger on “2,” the middle finger on “3,” and the index finger on “4” (Figure 1). The hand was covered with an opaque cloth to remove visual feedback. The task was structured into training and test blocks. A block was defined as a 30 s time-period during which the participant repeatedly typed the five-element sequence without pause or interruptions. The number of sequences a participant completed per block was not limited or restricted by any factors induced by the researcher. The five-element sequence “4–1–3-2-4” remained visible on the screen, and participants were instructed to type it “as fast and as accurately as possible.” The font color changed after every five key presses, signaling a new sequence. A completed sequence was defined as five consecutive key presses, and the sequence was considered correct if the five-element sequence was typed in the order of ‘4–1–3-2-4’. Each block was followed by 30 s of rest during which a fixation cross was displayed on the monitor (Karni et al., 1998; Walker et al., 2002; Nishida and Walker, 2007; Karni, 1995) (Figure 1). Because the sequence remained continuously visible during a block and no awareness probes were administered, we deliberately avoided describing this as implicit learning. Instead, the task is better interpreted as sequence-cued, proprioceptively executed skill refinement (Shmuelof et al., 2012).

**Figure 1:**
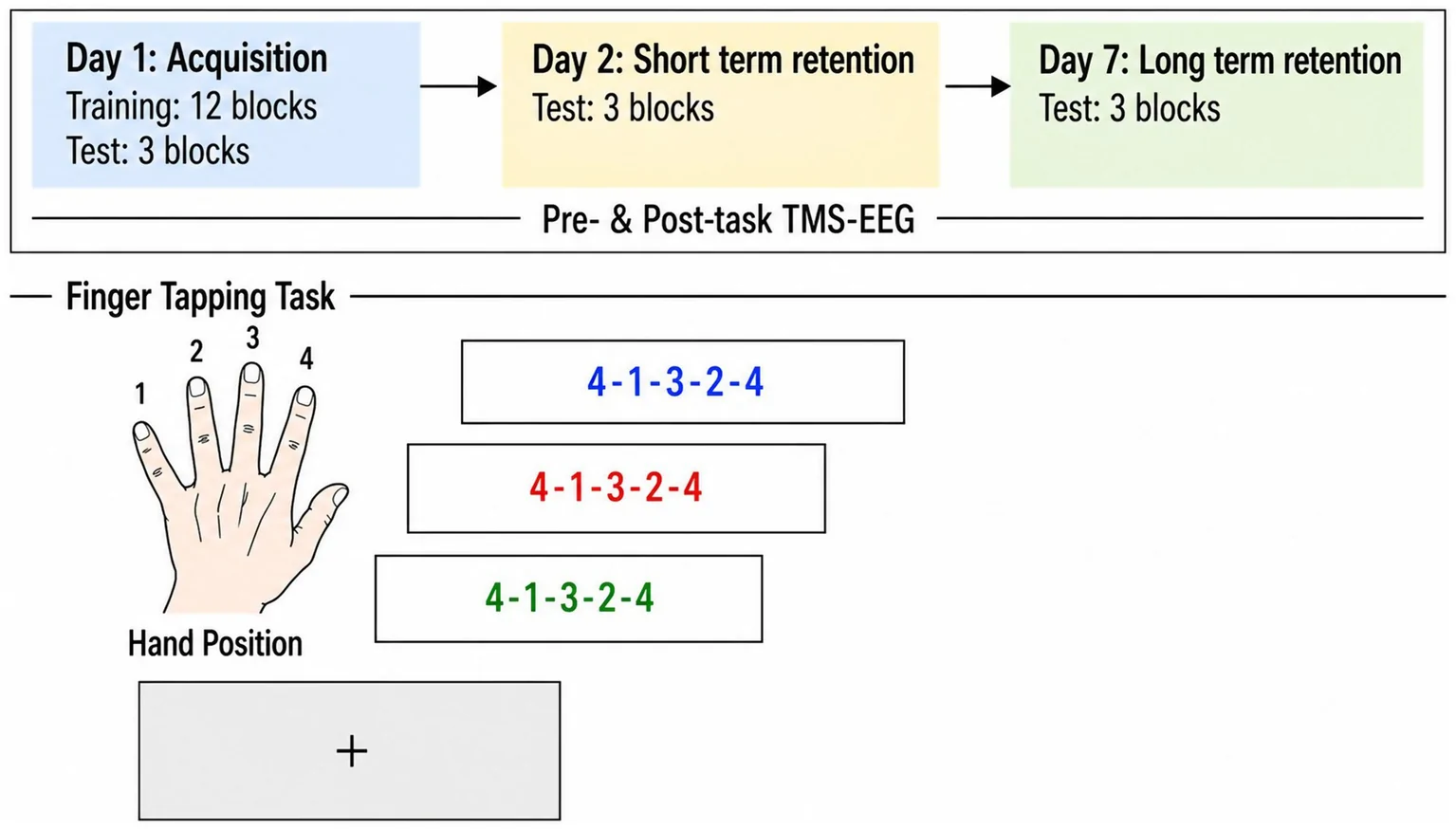
Experimental design and sequential finger-tapping task. Participants completed three sessions: Day 1, skill acquisition (12 training blocks + 3 test blocks); Day 2, short-term retention (3 test blocks), and Day 7, long-term retention (3 test blocks). TMS-EEG was acquired pre-task and post-task at each session. The task was performed with the non-dominant hand using the five-element sequence 4–1–3-2-4. Hand position: 1, little finger; 2, ring finger; 3, middle finger; 4, index finger. During a 30 s block colored sequence displays indicate the visual tapping cue. The fixation cross display indicates 30 s rest.

Day 1 comprised 12 training blocks followed by 3 test blocks. The test blocks were identical in timing and task demands compared to the training blocks; the only difference was that participants were told that their performance would be evaluated. Day 2 and 7 each comprised only three test blocks, identical to the test blocks of Day 1. Task execution was quantified using four measures: performance rate (PR), calculated as the total number of complete sequences in each 30 s block, including both correct and incorrect sequences; accuracy (ACC), defined as the total number of correct sequences per block, and calculated as total number of sequences completed per 30 s block (PR)—total number of incorrect sequences per 30 s block:


$$\mathrm{ACC}=\mathrm{PR}-\text{Tota}l_{\text{incorrect}/30s}$$

Error rate (ER), defined as the proportion of incorrect sequences relative to all attempted sequences, and calculated as total number of incorrect sequences per 30 s block / total number of sequences completed per 30 s block (PR);


$$\mathrm{ER}=\frac{\text{Tota}l_{\text{incorrect}/30s}}{\mathrm{PR}}$$

And inverse efficiency score (IES) was calculated as the estimated time per completed sequence, calculated as 30 s divided by the number of completed sequences within each block (PR), and adjusted for the proportion of correct responses:


$$\mathrm{IES}=\frac{30/\mathrm{PR}}{1-\mathrm{ER}}$$

In the IES equation, 30/PR represents the average seconds required per completed sequence, and 1-ER represents the proportion of completed sequences that were correct. Given these definitions, the IES is algebraically equivalent to the time required per correct sequence. Thus, IES was expressed in seconds (s) per accuracy-adjusted sequence, with lower values indicating more efficient performance (Table 1).

**Table 1:** Sequential motor learning task performance measurement parameters.

Variable	Formula	Units
Performance rate (PR)	Total number of complete sequences per 30 s block	Sequences/30 s
Accuracy (ACC)	$\mathrm{ACC}=\mathrm{PR}-\text{Tota}l_{\text{incorrect}/30s}$	Sequences/30 s
Error rate (ER)	$\mathrm{ER}=\frac{\text{Tota}l_{\text{incorrect}/30s}}{\mathrm{PR}}$	Dimensionless (range: 0–1)
Inverse efficiency score (IES)	$\mathrm{IES}=\frac{30/\mathrm{PR}}{1-\mathrm{ER}}$	s/accuracy-adjusted sequence

The four performance measures used to quantify the behavioral data.

Although IES combines completion rate with the proportion of correct sequences, thereby incorporating the effect of error rate, in this task it is mathematically equivalent to the inverse of accuracy-adjusted throughput. Accordingly, IES was interpreted alongside the separate PR, ACC, and ER measures because changes in the composite score may reflect changes in the individual performance measures or a combination thereof. At a fixed PR, higher error rates reduce the denominator (1-ER) and increase the IES, indicating lower efficiency. Because this task was continuous and self-paced, IES was used as a composite index of efficiency to guard against misleading interpretations when ER and accuracy changed in opposite directions (Reis et al., 2009; Shmuelof et al., 2012; Liesefeld and Janczyk, 2019).

### 2.5 Transcranial magnetic stimulation

At the start of each session, the RMT was determined from the left first dorsal interosseous (FDI) muscle using surface electromyography with Ag/AgCl electrodes in a belly-tendon configuration and a ground electrode over the ulnar styloid process. The right M1 hotspot was identified as the scalp position that elicited the largest and most reliable motor-evoked potentials (MEPs) in the FDI. The coil was placed on the EEG cap forming a 45° angle between the handle of the coil and the sagittal plane, with a posterior-to-anterior induced-current direction. Stimulation intensity started at 70% of the maximum stimulator output and was decreased in 2% increments until at least 5 of 10 pulses produced MEPs with a peak-to-peak amplitude ≥ 50 μV (King et al., 2026). The coil position was marked on the EEG cap for consistent placement across pre-task and post-task stimulation (Sondergaard et al., 2021).

Eighty biphasic single pulses were delivered at a fixed pulse interval of 0.25 Hz at 120% of the session-specific RMT. Participants were instructed to keep their eyes open, facial and hand muscles relaxed, and to avoid voluntary movement during stimulation. A pillow supported the head and neck, reducing movement and improving coil stability (Rossi et al., 2021).

### 2.6 Electroencephalography acquisition

EEG was recorded with a Waveguard 64-channel TMS-compatible cap (sintered Ag/AgCl electrodes, ANT Neuro) positioned according to the international 10–10 system (Oostenveld and Praamstra, 2001). Signals were amplified using an EEGO MyLab system (ANT Neuro), hardware filtered from DC to 2.08 kHz and digitized at 8 kHz. CPz served as online reference, and AFz as ground. Electrode impedance was maintained below 5 kΩ.

### 2.7 Auditory and somatosensory artifact mitigation

To reduce auditory and somatosensory contamination of TEPs, a multimodal suppression protocol was used (ter Braack et al., 2015; Conde et al., 2019; Mutanen et al., 2020). A 5-mm foam spacer was placed between the coil and the EEG cap to damp scalp vibrations and reduce bone-conducted sound. Participants wore in-ear earphones delivering individually adjusted white noise generated with the TMS Adaptable Auditory Control (TAAC) interface (Biabani et al., 2019), together with over-ear noise-cancelling earmuffs (King et al., 2026).

On Day 1, prior to the pre-task active TMS, 40 sham pulses were delivered with the coil held vertically above (0.5–1 cm) the marked right M1 location, using the same intensity and interstimulus interval as active TMS. Active TMS was delivered immediately after the sham pulses. The sham and active TMS-EEG data were collected in a single continuous EEG dataset. These sham trials were used during independent component analysis (ICA) to aid in the identification of auditory artifact components (King et al., 2026; Biabani et al., 2019). Sham data were not directly subtracted from active TMS data because subtraction can distort genuine cortical signals (Rogasch and Fitzgerald, 2013; King et al., 2026; Conde et al., 2019).

### 2.8 EEG data processing and TEP analysis

EEG preprocessing was performed in MATLAB R2025b using EEGLAB (v2025.1.0) (Delorme and Makeig, 2004) and the TMS-EEG Signal Analyzer (TESA) toolbox (Mutanen et al., 2020; Rogasch et al., 2017). Data were imported into EEGLAB and the TESA pipeline was followed as described by Rogasch et al. (2017) (Rogasch et al., 2017). EEGLAB’s default settings were used for the automated functions described below unless otherwise stated. The Day 1 pre-task TMS-EEG datasets, containing 40 sham and 80 active TMS pulses, were analyzed as a single continuous dataset. The 40 sham pulses were segmented and merged with the Day 1 post-task, Day 2 pre and post-task, and Day 7 pre and post-task TMS-EEG datasets before commencing the preprocessing pipeline. Bad electrodes were removed using the EEGLAB automatic channel rejection function. Afterwards, the data were epoched from −1,000 ms to +1,000 ms around each TMS pulse, and the baseline correction range was set from −1,000 to 0 ms. Following segmentation and merging, each dataset comprised 120 epochs, of which the first 40 originated from the Day 1 sham pulses and the remaining 80 from the corresponding active TMS recordings. Bad epochs were identified and removed using the automated EEGLAB “reject data epochs” function. Across all sessions and conditions, none of the 40 sham epochs were rejected, and ≥ 70 of the 80 active TMS epochs were retained in every dataset. Thus, all the datasets entered ICA with ≥110 of 120 epochs (≥ 91.7%) retained. The data were downsampled to 1 kHz. The immediate TMS pulse artifact from −2 to 15 ms relative to the TMS pulse was removed and linearly interpolated using the TESA plugin. A 1–80 Hz band-pass filter and a 50 Hz notch filter were applied. Large-amplitude artifacts, including TMS-evoked muscle, electrical, movement, and auditory artifacts, were identified and automatically removed using the TESA FastICA and Component Classification functions. The autodetection of artifact components was set as follows: TMS-evoked muscle activity detection threshold 8, with target times of 11–30 ms; eye-blink detection threshold 2.5, using electrodes Fp1 and Fp2; lateral eye-movement detection threshold 2, using electrodes F7 and F8; persistent muscle activity detection threshold −0.31, with included frequencies of 7–70 Hz and excluded frequencies of 48–52 Hz; and the electrode-noise detection threshold 4. The sham data assisted with the identification of auditory artifacts by increasing the independent component (IC) weight (Biabani et al., 2019). The autodetected components were reviewed by the researcher before the artifact components were removed; approximately 87% of the total ICs were retained. Channels removed during the channel quality check were interpolated, and the data were re-referenced to the average. This was followed by extraction of the region of interest (ROI) and identification of the peaks of interest. The right-M1 ROI was defined as C2, FC2, C4, and FC4. Signals from these four electrodes were averaged before peak extraction, and TEP amplitudes were quantified within canonical latency windows as N45 (35–55 ms), N100 (80–120 ms), P180 (160–200 ms), and N280 (260–300 ms) (Komssi and Kähkönen, 2006; Lioumis et al., 2009). Peak amplitudes were exported for statistical analysis.

### 2.9 Statistical analysis

Statistical analysis was performed using IBM SPSS Statistics (Version 31.0.1). Behavioral slopes for PR, ACC, ER, and IES across the Day 1 twelve training blocks were evaluated separately using one-sample Wilcoxon signed-rank tests to determine whether each measure changed systematically across training. Because these measures differ in scale and, for ER, dimensionality, raw slope magnitudes were not compared directly across behavioral metrics. Spearman correlations were used to assess relationships among slopes.

For each participant, within-phase learning slopes were estimated for early Day 1 training, Day 1 testing (TD1), Day 2 testing (TD2), and Day 7 testing (TD7). These slopes were compared pairwise using paired Wilcoxon signed-rank tests: (i) early Day 1 training, defined as the first three training blocks, versus Day 1 testing (TD1); (ii) Day 1 testing (TD1) versus Day 2 testing (TD2), representing the short 24-h retention interval; and (iii) Day 2 testing (TD2) versus Day 7 testing (TD7), representing the longer 5-day retention interval. Absolute values of approximately 0.10, 0.30, and 0.50 were interpreted as small, medium, and large effects, respectively. One participant had incomplete analyzable Day 1 behavioral data for which an ACC, ER, and IES slope could not be determined. Model 1 analyses used *n* = 16 and interval-level analyses *n* = 17.

Model 1 tested whether baseline TEP amplitude or acute learning-rate TEP modulation was associated with individual differences in rapid Day 1 motor learning. Separate component-specific multiple linear regression models were fitted for N45, N100, P180, and N280 (*n* = 16). The dependent variable was Day 1 IES learning slope calculated across the 12 training blocks. For each component, baseline TEP amplitude (Pre) was defined as the amplitude measured immediately before the Day 1 finger-tapping learning session, whereas Post represented the corresponding amplitude measured immediately after learning. Acute TEP change was calculated as:


$$\mathrm{dTE}P_{i}=\mathrm{Pr}e_{i}-\mathrm{Pos}t_{i}$$

Because baseline amplitude and raw dTEP were correlated, dTEP was orthogonalized with respect to baseline amplitude by regressing dTEP on Pre and retaining the residual (dTEP*_i_*^⊥^). This allowed the acute TEP change term to represent learning related modulation independent of baseline TEP amplitude and reduced collinearity between the two predictors. Each component-specific model was:


$$\mathrm{LB}\_\mathrm{IES}\_\text{Slop}e_{i}=\beta_{0}+\beta_{1}\mathrm{Pr}e_{i}+\beta_{2}\mathrm{dTE}P_{⊥i}+\varepsilon_{i}$$

where *β*_1_ represents the association between baseline TEP amplitude and Day 1 learning and *β*_2_ represents the association between baseline-independent acute TEP change and Day 1 learning. Effect magnitude was quantified using partial correlation coefficients (r_partial_), with absolute values of approximately 0.10, 0.30, and 0.50 interpreted as small, medium, and large, respectively.

Model 2 tested whether interval-specific baseline TEP amplitude and acute TEP modulation were associated with subsequent IES performance and whether the association with acute TEP modulation differed between the 24-h (TD1 → TD2) interval and the 5-day (TD2 → TD7) interval. Separate repeated-measures linear mixed models were fitted for N45, N100, P180, and N280 (*n* = 17; 32 valid interval-level observations). Participants contributed up to two interval-level observations. For the TD1 → TD2 interval, the outcome was the TD2 IES Slope and prior performance was the TD1 IES slope; for TD2 → TD7, the outcome was the TD7 IES slope and prior performance was the TD2 IES slope. Thus, the prior IES slope adjusted for the participant’s behavioral state entering each interval. Physiological predictors were assigned according to the beginning of each interval: Day 1 TEP measures were assigned to TD1 → TD2 and Day 2 TEP measures to TD2 → TD7. Pre represented the pre-task TEP amplitude on the corresponding day, and acute TEP change was calculated as:


$$\mathrm{dTE}P_{\mathrm{ij}}=\mathrm{Pr}e_{\mathrm{ij}}-\mathrm{Po}st_{\mathrm{ij}}$$

Unlike Model 1, dTEP was not orthogonalized in Model 2. The 24-h interval was specified as the reference category. The component-specific model was:


$$\begin{array}{l} Y_{\text{Future},\mathrm{ij}}=\beta_{0}+\beta_{1}\text{Interva}l_{\mathrm{ij}}+\beta_{2}Y_{\text{prior},\mathrm{ij}}\\ +\beta_{3}\mathrm{Pr}e_{\mathrm{ij}}+\beta_{4}\mathrm{dTE}P_{\mathrm{ij}}+\beta_{5}(\text{Interva}l_{\mathrm{ij}}\times \mathrm{dTE}P_{\mathrm{ij}})+\varepsilon_{\mathrm{ij}} \end{array}$$

Where *i* indexes participant and *j* indexes interval. Y_Future_ represents the subsequent IES slope and Y_prior_ the IES slope entering the interval. With the 24-h interval as the reference, *β*_4_ represents the dTEP association during TD1 → TD2, whereas *β*_5_ represents the difference in this association between the 5-day and 24-h intervals. No continuous predictors were centered or standardized.

Within-participant repeated observations were modeled using a diagonal covariance structure, allowing interval-specific residual variances while specifying zero residual covariance between intervals. Models were estimated using restricted maximum likelihood with Satterthwaite denominator degrees of freedom. Interval-specific simple slopes were estimated for significant Interval × dTEP interactions. No prospective power calculation was performed specifically for these exploratory component-specific mixed models. Given the available sample of 17 participants contributing 32 valid interval-level observations, inference emphasized coefficient estimates and 95% confidence intervals, together with FDR correction and participant-level sensitivity analyses. Model-fit indices, interval-specific residual variance estimates, and convergence diagnostics are reported.

To address multiplicity across the component-specific physiological hypotheses, Benjamini-Hochberg false discovery rate (FDR) correction was applied separately to the two model families. For Model 1, eight TEP-related tests were corrected together (Pre and orthogonalized dTEP for each of four components). For Model 2, 12 TEP-related Type III fixed-effect tests were corrected together (Pre, dTEP, and Interval × dTEP for each component). Interval and prior-performance terms were not included because they were behavioral/model covariates rather than competing TEP hypotheses. Both raw *p*-values and FDR-adjusted q-values are reported.

Because the nominal P180 association in Model 2 was the only physiological finding reaching the unadjusted significance threshold, additional sensitivity analyses were performed for this component. Leave-one-participant-out analyses repeated the identical P180 model after sequentially excluding each participant. A 5,000-replicate participant-level cluster bootstrap additionally resampled participants with replacement while retaining all available interval observations belonging to each sampled participant. Bootstrap percentile and bias-corrected and accelerated (BCa) 95% confidence intervals were calculated for the 24-h P180 dTEP slope and Interval × dTEP interaction.

## 3 Results

### 3.1 Behavioral dynamics during day 1 acquisition

Across the 12 training blocks on Day 1, participants showed significant within-session changes in task execution. For each behavioral measure, a participant-level slope was estimated across the 12 blocks and tested against zero using a one-sample Wilcoxon signed-rank test. All four differed significantly from zero (all *p* < 0.001) (Supplementary Table S1 Panel A).

To assess whether the change in the composite IES measure was reflected in its individual behavioral components, PR and ER were also examined separately as sensitivity analyses. Performance rate (PR) showed a positive slope across training (median slope = 0.12 sequences/30s, *Z* = −3.62), indicating more completed sequences per block. ACC also showed a positive slope across training (median slope = 0.31, *Z* = −3.52). ER showed a median slope close to zero (−0.004, *Z* = −3.52). Nevertheless, the Wilcoxon signed-rank test was significant (*Z* = −3.52, *p* < 0.001), indicating that across the group the distribution of ER slopes was consistently skewed toward small negative values. Thus, most participants showed a modest reduction in errors across training. IES also decreased across training, indicating improved accuracy-adjusted efficiency, with a median slope of −1.49 s/accuracy-adjusted sequence (*Z* = −3.52) (Supplementary Table S1 Panel A).

Because PR, ACC, ER, and IES differ in scale and dimensionality, their raw slope magnitudes were not interpreted comparatively. Instead, each measure was considered separately with respect to the direction and consistency of change across training. The IES findings therefore reflect change in the composite performance-accuracy measure and should not be interpreted as indicating a greater magnitude of change than PR, ACC, or ER. Spearman correlations among Day 1 slopes showed that changes in PR were not associated with changes in ACC or IES (all *ρ* < 0.10, *p* > 0.70), suggesting that inter-individual variation in PR was dissociable from changes in precision. By contrast, participants who showed greater improvement in accuracy also tended to show larger reductions in error rate (*ρ* = −0.691, *p* = 0.003) and greater efficiency (*ρ* = −0.503, *p* = 0.047). The positive association between ER and IES slopes (*ρ* = 0.471, *p* = 0.066) did not reach statistical significance; the direction is nonetheless consistent with the IES formula, as participants whose error rates decreased more across blocks also tended to show better efficiency.

To determine whether the observed efficiency improvement reflected a genuine change in the performance–accuracy function rather than simple slowing, session-level mean IES values, averaged across test blocks within each session, were compared across training and test phases using Wilcoxon signed-rank tests. Median IES decreased from 12.02 s/accuracy-adjusted sequence (IQR = 11.58) during early training (blocks 1–3) to 2.65 s/accuracy-adjusted sequence (IQR = 6.43) during late training (blocks 10–12), a median paired reduction of 9.37 s/accuracy-adjusted sequence (W = 21.00, Z = −2.43, *p* = 0.015, *r* = 0.61, effective n = 16). Median IES also decreased from early training to the Day 1 test session (TD1), from 12.02 s/accuracy-adjusted sequence (IQR = 11.58) to 2.50 s/accuracy-adjusted sequence (IQR = 5.45), a median paired reduction of 9.52 s/accuracy-adjusted sequence (*W* = 29.00, *Z* = −2.02, *p* = 0.044, *r* = 0.50, effective *n* = 16). Together, these medium-to-large effects demonstrate a change in composite IES measures over the course of Day 1 (Figure 2; Table 2; Supplementary Table S1 Panel B).

**Figure 2:**
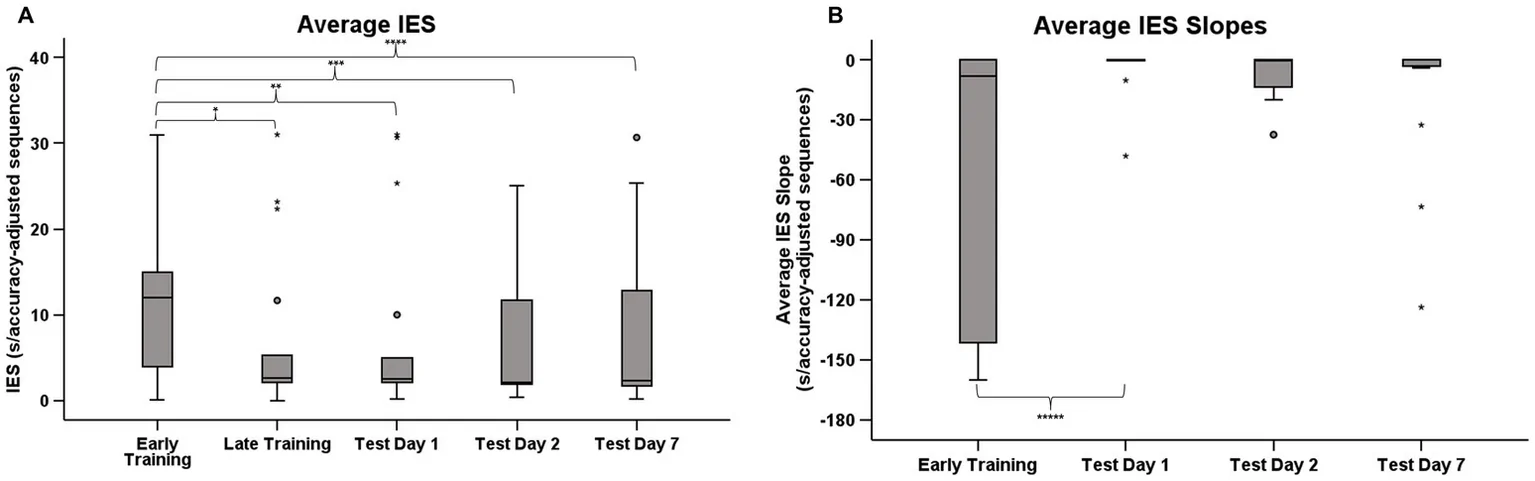
Stability of within-session IES slopes across acquisition and retention sessions. **(A)** Mean IES across early training (blocks 1–3), late training (blocks 10–12), and test sessions (TD1, TD2, TD7). IES decreased from early to late training (*p = 0.015), and from early training to TD1 (**p = 0.044), TD2 (***p = 0.031), and TD7 (****p = 0.028). There were no significant differences between late training and test sessions or among the test sessions (all *p* ≥ 0.407). **(B)** Within-session IES slopes were steeper during early Day 1 training than at TD1 (*****p = 0.005) and remained stable across TD1, TD2, and TD7.

**Table 2:** Behavioral metrics (median ± IQR) for each phase.

Session/phase	PR (sequences/30s)	ACC (sequences/30s)	ER	IES (s/accuracy-adjusted sequence)
Day 1—training (blocks 1–12) (*n* = 17)	12.33 ± 4.87	9.08 ± 8.08	0.25 ± 0.42	5.75 ± 9.02
Day 1—early training (blocks 1–3) (*n* = 17)	8.67 ± 5.83	6.67 ± 5.67	0.33 ± 0.47	12.02 ± 11.58
Day 1—late training (blocks 10–12) (*n* = 17)	14.33 ± 5.83	11.67 ± 10.17	0.22 ± 0.40	2.65 ± 6.43
TD1 (Day 1 test session) (*n* = 17)	14.67 ± 5.17	12.00 ± 9.50	0.21 ± 0.73	2.50 ± 5.45
TD2 (Day 2 test session) (*n* = 17)	16.00 ± 6.33	14.33 ± 13.00	0.08 ± 0.64	2.16 ± 11.32
TD7 (Day 7 test session) (*n* = 17)	16.33 ± 9.00	13.33 ± 13.83	0.06 ± 0.76	2.37 ± 11.42

Behavioral performance metrics absolute values during the training and test phase. Performance rate (PR), accuracy (ACC), error rate (ER), and inverse-efficiency score (IES) are reported as group median ± IQR.

To assess whether efficiency gains persisted beyond Day 1, session-level mean IES was compared across time points using Wilcoxon signed-rank tests. The median IES was lower at TD2 (2.16 s/accuracy-adjusted sequence, IQR = 11.32) than during early training (12.02 s/accuracy-adjusted sequence, IQR = 11.58; W = 31.00, *Z* = −2.15, *p* = 0.031, *r* = 0.52, effective n = 17), and a similar pattern was observed for early training versus TD7 (2.37 s/accuracy-adjusted sequence, IQR = 11.42; W = 30.00, *Z* = −2.20, *p* = 0.028, *r* = 0.53, effective n = 17). In contrast, late training mean IES did not differ significantly from TD1, TD2, or TD7 (all *p* > 0.50, r ≤ 0.17), indicating that efficiency at the end of Day 1 was maintained across later test sessions (Figure 2; Table 2; Supplementary Table S1 Panel B).

Comparisons among the three test sessions confirmed stability: mean IES did not differ between TD1 and TD2 (*p* = 0.407), TD1 and TD7 (*p* = 0.435), or TD2 and TD7 (*p* = 0.586). Together, these findings corroborate the slope-based analyses: participant’s demonstrated efficiency gain during Day 1, and test-session efficiency remained stable across both retention intervals (Figure 2; Supplementary Table S1 Panel B).

### 3.2 Transition from training to test, retention, and long-term interval change

To test whether within-session dynamics transferred from early training to the Day 1 test session (TD1), slopes from the first three training blocks were compared with those from the three TD1 test blocks. IES decreased more steeply during early training (median IES slope = −8.17 s/accuracy-adjusted sequence, IQR = 147.46) than during TD1 (median IES slope = −0.12 s/accuracy-adjusted sequence, IQR = 0.41) (W = 109.00, *Z* = 2.78, *p* = 0.005, *r* = 0.72; effective *n* = 15) (Figure 2B). This pattern indicates a greater rate of within-session efficiency improvement during early training and relatively little additional change during TD1. Slopes for PR, ACC, and ER did not differ significantly between early training and TD1.

We next examined the stability of within-session dynamics across the subsequent test sessions. No significant differences were found between TD1 and TD2 for any of the four metrics (all *p* ≥ 0.156), a pattern consistent with similar within-session slope profiles across the 24-h interval. When comparing TD2 with TD7, PR and IES slopes did not differ significantly, and ACC showed no statistically significant difference. In contrast, ER slopes differed between TD2 and TD7 (*W* = 78.00, *Z* = 2.27, *p* = 0.023, *r* = 0.61, effective *n* = 14), with more negative slopes and greater variability at TD2 than at TD7. These findings suggest that most aspects of within-session change were similar across the 5-day interval, whereas the pattern of ER change differed between TD2 and TD7. The slope-based analyses characterize the structure of within-session change rather than absolute performance levels and therefore do not, on their own, establish retention or long-term stabilization of the learned task.

Grand-average right-M1 waveforms and component amplitudes were examined descriptively before and after the finger-tapping task on each day. P180 amplitudes shifted towards more positive values after the task on Day 1 and 2, whereas pre and post-task amplitudes were similar on Day 7 (Figure 3). These pre-to-post contrasts are presented to illustrate the observed waveform patterns. The inferential analysis below tested associations between TEP measures and behavioral rather than standalone group-level pre-to-post effects.

**Figure 3:**
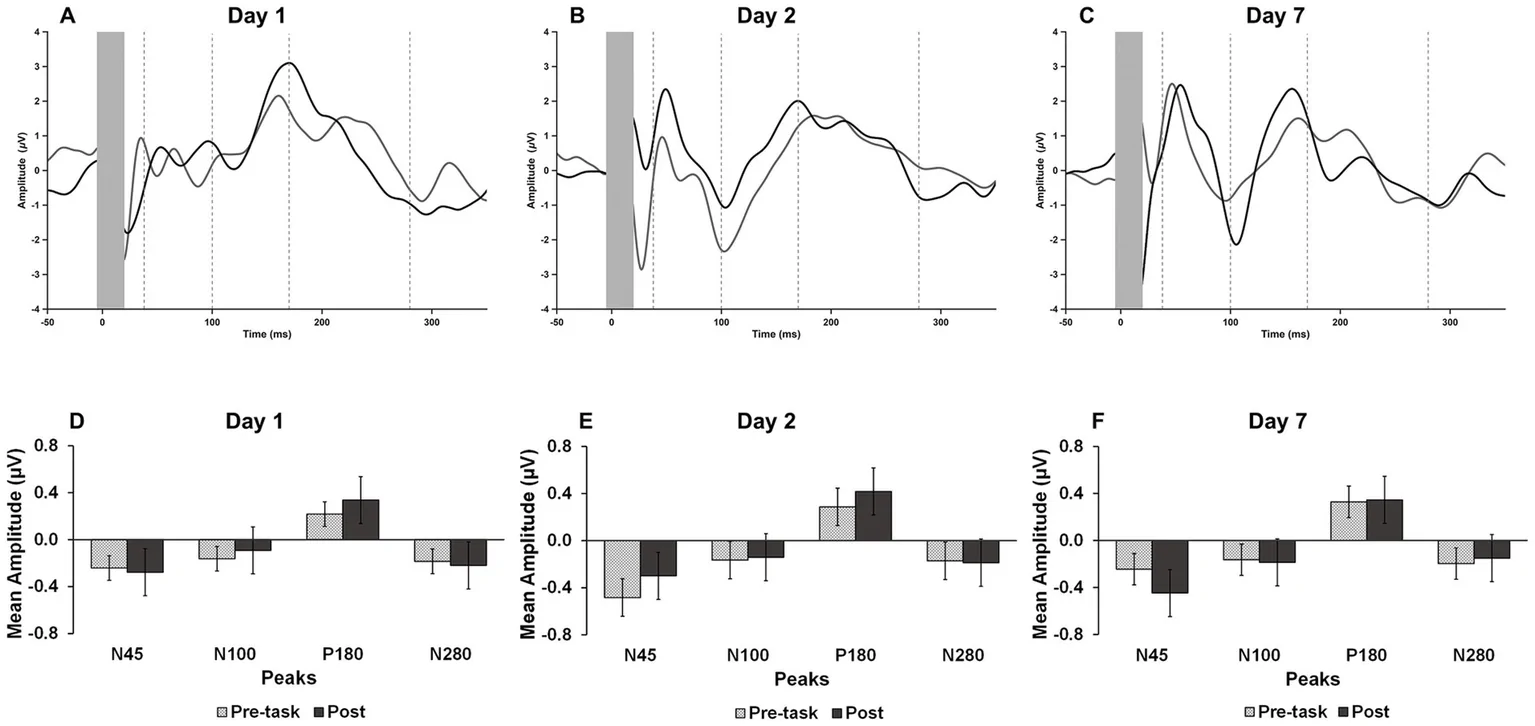
Grand average TMS-evoked potential over right M1 before and after task performance. Panels **(A–C)** show grand average TEPs from the right-M1 ROI (mean of C2, FC2, C4, and FC4) pre-task (light grey) and post-task (black) on Day 1 **(A)**, Day 2 **(B)**, and Day 7 **(C)**. Dotted vertical lines indicate canonical component latencies (N45, N100, P180, N280). Panels **(D–F)** show mean (±SEM) pre and post-task amplitudes per component per day.

### 3.3 TEP predictors of learning: model 1

Model 1 examined associations between baseline TEP amplitude, acute learning-related TEP modulation, and rapid Day 1 motor learning (*n* = 16). Baseline amplitude and raw dTEP were correlated, particularly for N45 (*r* = 0.835; VIF = 3.31 in the initial non-orthogonalized model), supporting orthogonalization of dTEP with respect to baseline amplitude.

Model fit was low across components (*R*^2^ = 0.001–0.243), and none of the four component-specific regression models was statistically significant. Neither baseline TEP amplitude nor orthogonalized acute TEP change was significantly associated with initial efficiency improvement; observed partial correlations were small to moderate in magnitude (r_partial_ ≤ 0.49). The lowest unadjusted predictor *p*-value was observed for baseline N45 amplitude (raw *p* = 0.063), but this association was substantially attenuated after correction for the eight Model 1 TEP-related comparisons (*q* = 0.445). No Model 1 TEP predictor remained significant following Benjamini-Hochberg FDR correction (all *q* ≥ 0.445).

Thus, within the available sample size and measurement precision, neither baseline right-M1 TEP amplitude nor baseline-independent acute learning-related TEP modulation showed statistically reliable evidence of association with inter-individual differences in rapid acquisition. Complete model coefficients and FDR-adjusted results are provided in Supplementary Table S2.

### 3.4 TEP predictors of learning: model 2

Model 2 examined whether interval-specific baseline TEP amplitude and acute TEP modulation were associated with subsequent IES performance and whether the association with acute modulation differed between the 24-h (TD1 → TD2) and 5-day (TD2 → TD7) intervals. Separate component-specific repeated-measures models were fitted for N45, N100, P180, and N280 (*n* = 17; 32 valid interval-level observations). All final models were estimable and converged without warnings under the pre-specified diagonal (DIAG) covariance structure for repeated observations. Model-fit indices, interval-specific residual variance estimates, and convergence information for all four component-specific models are provided in Supplementary Table S3. For N45, N100, and N280, none of the TEP-related fixed effects reached the unadjusted significance threshold. Moreover, no baseline TEP, dTEP, or Interval × dTEP effect for any component remained statistically significant after Benjamini-Hochberg correction across the 12 Model 2 TEP-related tests (all *q* ≥ 0.204) (Supplementary Table S4).

P180 showed a nominal interval-dependent association with subsequent IES slope before multiple-comparison correction. During the 24-h reference interval, larger P180 dTEP values were associated with more “negative” subsequent IES slope [B = −281.07, 95% CI (−519.28, −42.86), raw *p* = 0.024; *q* = 0.204] (Figure 4). The P180 dTEP association differed between intervals, as indicated by the positive Interval × dTEP interaction [B = 253.12, 95% CI (15.27, 490.97), raw *p* = 0.039; *q* = 0.204]. The corresponding simple slope during the 5-day interval was not statistically significant [B = −27.95, 95% CI (−90.64, 34.74), *p* = 0.354] (Figure 4). Thus, the negative P180 dTEP association was more pronounced during the 24-h interval than during the 5-day interval. Because dTEP was calculated as Pre − Post, its sign represents a change in the recorded, signed P180 voltage. It should not be interpreted directly as an increase or decrease in cortical activity without additional assumptions about TEP polarity and its physiological meaning.

**Figure 4:**
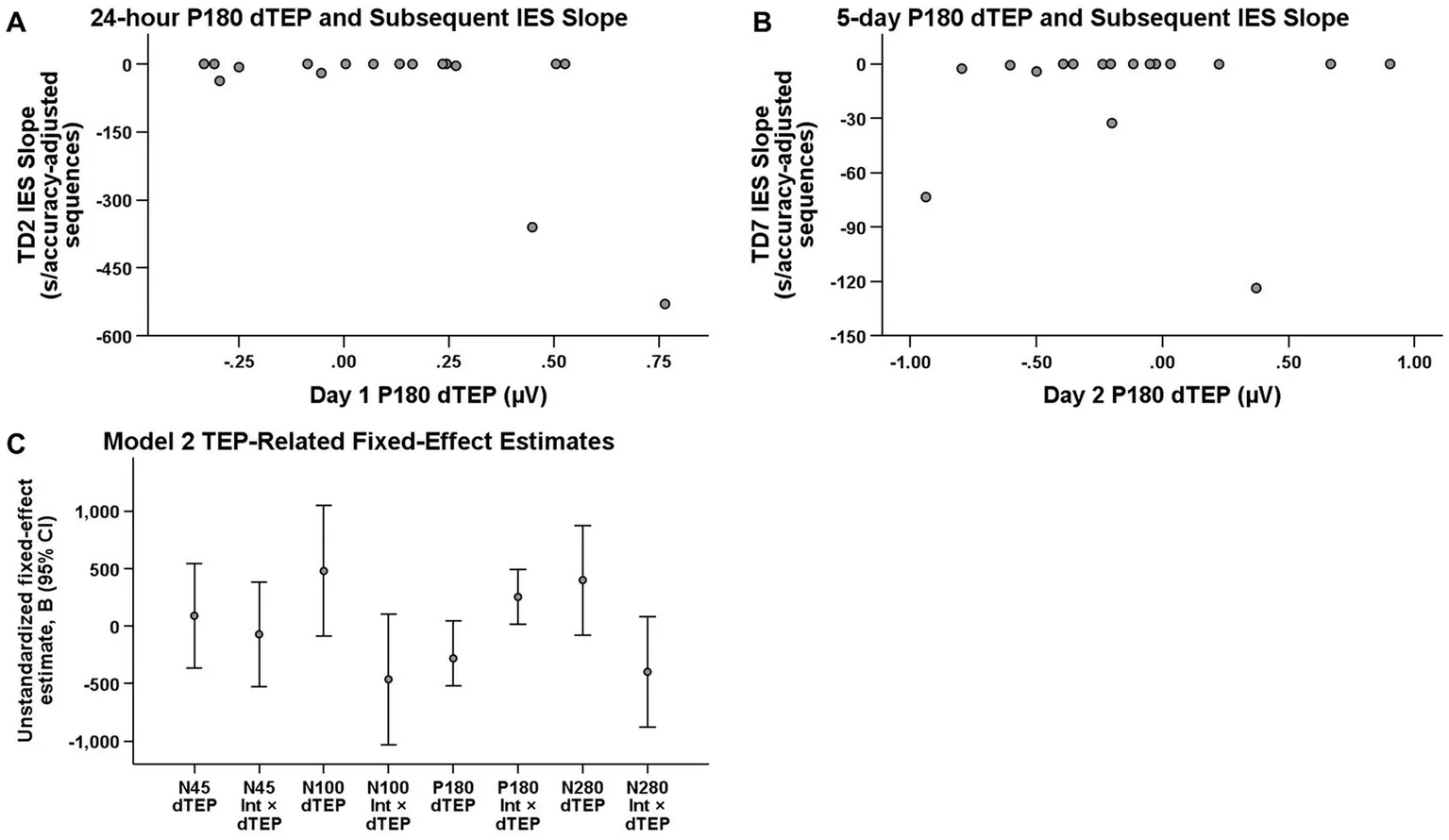
P180 dTEP and subsequent behavioral performance across intervals. **(A,B)** show raw interval-level observations relating P180 dTEP to subsequent IES slope over the 24-h (TD1 → TD2) and 5-day (TD2 → TD7) intervals, respectively (*n* = 17 per interval). Model 2 included 32 valid interval-level observations (15 for the 24-h interval and 17 for the 5-day interval) after complete-case exclusion for the model covariates. No regression lines are shown because the inferential analyses were based on the covariate-adjusted mixed-effects Model 2 rather than unadjusted bivariate regressions. No observations fall outside the displayed axis limits. **(C)** shows the unstandardized Model 2 fixed-effect estimates (*B*) and 95% confidence intervals for dTEP and Interval × dTEP for N45, N100, P180, and N280.

The statistical evidence for the P180 findings weakened after accounting for multiple comparisons. Neither the Type III dTEP effect (raw *p* = 0.018; *q* = 0.204) nor Interval × dTEP interaction (raw *p* = 0.039; *q* = 0.204) survived multiplicity correction. Participant-level sensitivity analyses indicated that the nominal associations were sensitive to participant-level influence. Across the 17 leave-one-participant-out analyses, the 24-h dTEP coefficient remained negative, the interaction coefficient remained positive, and all models converged. However, exclusion of Participant 16 substantially attenuated both the 24-h association [B = −99.88, 95% CI (−292.88, 93.11), *p* = 0.283] and interaction [B = 76.77, 95% CI (−116.42, 269.96), *p* = 0.406]. These results indicate that the direction of the P180 associations was consistent across resamples, but their magnitude and nominal statistical evidence were influenced appreciably by Participant 16. Participant-level bootstrap analyses provided convergent evidence of limited inferential robustness. Across 5,000 cluster-bootstrap replicates, the 95% percentile CI for the 24-h P180 dTEP coefficient was [−545.08, 4.11] and the BCa 95% CI was [−643.72, 1.35]. For the Interval × dTEP interaction, the corresponding intervals were [−35.25, 509.99] and [−16.87, 603.16]. All intervals included zero. Together with the leave-one-participant-out analysis, these findings indicate that the P180 associations were directionally consistent but sensitive to participant-level influence and sampling variability.

Collectively, the primary model provided nominal evidence of an interval-dependent P180 dTEP association with subsequent motor-performance change. The association appeared stronger during the 24-h interval than during the 5-day interval. However, neither the dTEP effect nor the Interval × dTEP interaction survived FDR correction, and both were sensitive to participant-level influence and bootstrap resampling. The P180 finding is therefore interpreted as exploratory, directionally consistent but statistically non-robust association rather than as evidence of a confirmed physiological correlate of subsequent motor performance. Complete model coefficients and FDR-adjusted results are provided in Supplementary Table S4.

## 4 Discussion

### 4.1 Summary of main findings

This study examined whether baseline TEP amplitudes and pre-to-post TEP changes over right M1 were associated with rapid acquisition of an explicit motor sequence task performed with the non-dominant left hand, across Day 1 acquisition and subsequent 24-h and 5-day intervals. Several aspects of within-session behavioral dynamics were broadly maintained across later sessions. Baseline TEP amplitudes and acute pre-to-post changes were not associated with rapid Day 1 acquisition, whereas late P180 change showed an exploratory interval-dependent association with subsequent efficiency change, with a stronger association over 24 h than 5 days. Because P180 was not a pre-specified primary finding, this result should be interpreted cautiously and requires replication.

### 4.2 Behavioral interpretation

During Day 1, IES decreased, consistent with improved composite performance efficiency. This reduction should not be interpreted as a simple increase in tapping rate: PR and ACC showed a modest positive slope, and ER showed a median slope close to zero. Because IES is a derived measure calculated from PR and ER, the separate component slopes should not be treated as a direct decomposition of the IES slope. In this dataset, changes in PR were not associated with changes in IES, whereas greater accuracy-related improvement was associated with greater efficiency gains. Thus, the behavioral pattern is more consistent with an adjustment in composite efficiency than with an isolated increase in speed.

By TD1, within-session IES slopes were markedly flatter than during the earliest Day 1 practice blocks, indicating that participants entered the first test session with a relatively stable efficiency profile and required less within-session adjustment. IES slopes remained broadly stable across TD1, TD2, and TD7, and mean IES did not differ across the three test sessions. Together, these findings are consistent with retention of the observed efficiency profile. However, slope-based analyses describe the structure of within-session change rather than absolute performance levels, and ER slopes differed between TD2 and TD7. Thus, the stability was not uniform across all behavioral measures.

### 4.3 Primary N100 hypothesis: null association

The primary N100 hypothesis was not supported. Neither baseline N100 amplitude nor acute pre-to-post N100 change was associated with rapid Day 1 acquisition or subsequent interval-related changes in efficiency. Although some N100 effect estimates were moderate, the confidence intervals were wide and the associations did not reach statistical significance. This finding differs from the association reported by Taga et al. (2021), who examined baseline N100 amplitude during a robot-mediated forcefield adaptation task involving left-M1 stimulation and dominant-hand performance (Taga et al., 2021). The difference may reflect the task, stimulation site, hand used, or learning timescale. Therefore, the present findings suggest that N100-behavior relationships may depend on the specific motor-learning context rather than being uniformly present across tasks.

### 4.4 Laterality and measurement scope

The task was performed with the non-dominant left hand, and TEPs were recorded over the contralateral right M1. Although this approach targeted a less-practiced motor system, sequence learning with the non-dominant hand engages a broader network extending beyond the contralateral M1, including the left lateral premotor cortex, bilateral supplementary motor areas, and additional frontal and temporal regions (Grafton et al., 2002; Johnson et al., 2002). Therefore, the absence of an association between right-M1 TEP measures and rapid acquisition may partly reflect the limited spatial coverage of a single-site recording approach. These measures may not have captured the distributed neural processes contributing to early learning. This interpretation represents a possible measurement-related explanation rather than a direct test of the mechanisms underlying acquisition.

### 4.5 Exploratory P180 finding

The exploratory analysis suggested a nominal relationship between pre-to-post P180 TEP change and subsequent efficiency change. This nominal association appeared stronger over the 24-h interval than over the 5-day interval. This pattern did not survive FDR correction and was sensitive to participant-level influence and bootstrap resampling. Late TEP components such as P180 have been proposed to reflect broader cortico-cortical propagation or integrative network-level activity, but their specific biological basis remains uncertain (Vernet et al., 2013; Tremblay et al., 2019). Previous work showing that P180 amplitude can be altered by interventions such as theta-burst stimulation indicates that this component is sensitive to changes in cortical state but does not establish a specific underlying mechanism (Carrette et al., 2025).

The nominally stronger association over the 24-h interval than over the 5-day interval may reflect time-dependent differences in the relationship between P180 TEP change and subsequent performance. It should therefore be interpreted as hypothesis-generating and not as evidence of P180 plasticity, offline reorganization, or a confirmed physiological correlate of learning. This finding should be interpreted cautiously because P180 was not a prespecified primary component, although multiple comparisons were adjusted. In addition, late TEP components may be more vulnerable than earlier components to residual auditory and somatosensory contamination (ter Braack et al., 2015; Conde et al., 2019; Biabani et al., 2019), and the biological basis of P180 remains less well established than that of the N100.

### 4.6 Limitations

Several limitations should be acknowledged. First, the sample size was modest, particularly for exploratory component-specific interval models. Although all models converged and participant-level sensitivity analyses were performed, the 17 participants contributing 32 valid interval-level observations provided limited information for estimating models containing multiple fixed effects and an interaction, thereby limiting the precision and stability of the estimates. Accordingly, these analyses should be considered exploratory and require confirmation in larger independent samples. Second, the task was explicit and sequence-cued, but awareness was not directly assessed. Third, although multiple steps were taken to reduce auditory and somatosensory contamination, residual sensory contributions to late TEP components cannot be fully excluded. Fourth, the session specific RMT and corresponding 120% RMT values in %MSO were not retained. Therefore, the absolute stimulation intensity across sessions was not directly evaluated. Finally, these findings apply specifically to a non-dominant-hand sequential tapping task in healthy adults and may not generalize directly to dominant-hand learning, adaptation paradigms, older adults, or clinical populations.

### 4.7 Future directions

Future studies should first replicate the interval-dependent association between pre-to-post P180 change and subsequent efficiency change in larger samples, with rigorous control of auditory and somatosensory confounds. Replication should also examine whether this association generalizes to dominant-hand performance and other motor-learning tasks. Studies that include sleep monitoring and assess multiple consolidation intervals, such as 4 to 6 h and 24 h, could determine whether the relationship between P180 change and subsequent performance varies as a function of interval duration (Wang et al., 2026).

## 5 Conclusion

Practice of an explicit motor sequence was accompanied by improved composite behavioral efficiency, and several aspects of within-session behavioral dynamics were broadly maintained across later test sessions. Baseline right M1 TEP amplitudes and acute pre-to-post changes were not associated with rapid Day 1 acquisition. An exploratory nominal association was observed between pre-to-post P180 TEP change and subsequent efficiency change, with a nominally stronger association over the 24-h interval than over the 5-day interval. This association did not survive FDR correction and was sensitive to participant-level influence and bootstrap resampling. Because it was not a pre-specified primary finding and late TEP components may be influenced by residual sensory contributions, it should be interpreted cautiously and not as evidence of P180 plasticity or a direct measure of cortical excitability. Larger replication studies using rigorous sensory control are needed before this association can be considered a candidate physiological correlate of subsequent efficiency change.

## Data Availability

The raw data supporting the conclusions of this article will be made available by the authors, without undue reservation.
