# Supplementary material for: Associations between pre-to-post TMS-evoked potential changes and motor sequence learning across retention intervals
Source: Front Hum Neurosci. 2026 Sep 23;20:1925178. doi: 10.3389/fnhum.2026.1925178 (PMC13642314; doi:10.3389/fnhum.2026.1925178)
Supplement: Supplementary file 1 [file Table_1.DOCX]

**Associations Between Pre-to-Post TMS-Evoked Potential Changes and Motor Sequence Learning Across Retention Intervals**

**Supplementary Table S1: Behavioral Wilcoxon signed-rank analyses**

**Panel A: One-sample Wilcoxon signed-rank tests of Day 1 behavioral slopes against zero**

| **Measure** | **N** | **Mean** | **Median** | **W** | | **Z** | | **Exact p** | **Effect size (r)** | **Mean Rank** | **Sum of Ranks** | **Ties (n)** |
| --- | --- | --- | --- | --- | --- | --- | --- | --- | --- | --- | --- | --- |
| PR | 17 | 0.150 | 0.123 | | 0.000 | -3.622 | <0.001 | | 0.878 | 9.000 | 153.000 | 0 |
| ACC | 16 | 0.469 | 0.313 | | 0.000 | -3.516 | <0.001 | | 0.879 | 8.500 | 136.000 | 0 |
| ER | 16 | -0.003 | -0.004 | | 0.000 | -3.516 | <0.001 | | 0.879 | 8.500 | 136.000 | 0 |
| IES | 16 | -2.351 | -1.492 | | 0.000 | -3.516 | <0.001 | | 0.879 | 8.500 | 136.000 | 0 |

**Supplementary Table S1 Panel A note:** W = Wilcoxon signed-rank test statistic; Z = standardized test statistic; r = effect size, calculated as r=∣Z∣/√N. Absolute values of approximately 0.10, 0.30, and 0.50 were interpreted as small, medium, and large effects, respectively. All tests were two-sided with α = 0.05. Analyses were conducted using one-sample Wilcoxon signed-rank tests. P-values were computed using exact inference. Mean rank and sum of ranks refer to the non-zero slopes contributing to the signed-rank test. Ties indicate slopes equal to the hypothesized value of zero. One participant had incomplete analyzable Day 1 behavioral slope data for ACC, ER, and IES rendering n = 16.

**Panel B: Pairwise Wilcoxon signed-rank comparisons of absolute mean IES across training and retention sessions**

| **Sessions** | **N** | **N_eff_** | **W** | **Z** | **Exact p  (2-tailed)** | **r** |
| --- | --- | --- | --- | --- | --- | --- |
| Early Training – Late Training | 17 | 16 | 21.00 | -2.430 | 0.015 | 0.61 |
| Early Training - TD1 | 17 | 16 | 29.00 | -2.017 | 0.044 | 0.50 |
| Late Training - TD1 | 17 | 16 | 65.00 | -0.155 | 0.877 | 0.04 |
| Early Training - TD2 | 17 | 17 | 31.00 | -2.154 | 0.031 | 0.52 |
| Late Training – TD2 | 17 | 16 | 55.00 | -0.672 | 0.501 | 0.17 |
| Early Training – TD7 | 17 | 17 | 30.00 | -2.202 | 0.028 | 0.53 |
| Late Training - TD7 | 17 | 17 | 68.00 | -0.402 | 0.687 | 0.10 |
| TD1 – TD2 | 17 | 17 | 59.00 | -0.828 | 0.407 | 0.20 |
| TD1 – TD7 | 17 | 17 | 60.00 | -0.781 | 0.435 | 0.19 |
| TD2 - TD7 | 17 | 17 | 65.00 | -0.544 | 0.586 | 0.13 |

**Supplementary Table S1 Panel B note:** W = Wilcoxon signed-rank test statistic; Z = standardized test statistic; N_eff_ = non-zero paired differences contributing to the Wilcoxon signed-rank test where zero-differences (ties) were excluded; r = effect size, calculated as $r=\mid Z\mid/\sqrt{N_{eff}}$. Absolute values of approximately 0.10, 0.30, and 0.50 were interpreted as small, medium, and large effect, respectively. All tests were two-sided with α = 0.05. Analyses were conducted using two-related-samples Wilcoxon signed-rank tests. P-values were computed using exact inference.

**Panel C: Ranks table for the pairwise Wilcoxon signed-rank comparisons of absolute mean IES across training and retention sessions**

| **Sessions** | **Positive Ranks** | | | **Negative Ranks** | | | **Ties (n)** |
| --- | --- | --- | --- | --- | --- | --- | --- |
|  | **N** | **Mean Rank** | **Sum of Ranks** | **N** | **Mean Rank** | **Sum of Ranks** |  |
| Early Training – Late Training | 14 | 8.21 | 115.00 | 2 | 10.50 | 21.00 | 1 |
| Early Training - TD1 | 13 | 8.23 | 107.00 | 3 | 9.67 | 29.00 | 1 |
| Late Training - TD1 | 7 | 10.14 | 71.00 | 9 | 7.22 | 65.00 | 1 |
| Early Training - TD2 | 13 | 9.38 | 122.00 | 4 | 7.75 | 31.00 | 0 |
| Late Training – TD2 | 11 | 7.36 | 81.00 | 5 | 11.00 | 55.00 | 1 |
| Early Training – TD7 | 14 | 8.79 | 123.00 | 3 | 10.00 | 30.00 | 0 |
| Late Training - TD7 | 11 | 7.73 | 85.00 | 6 | 11.33 | 68.00 | 0 |
| TD1 – TD2 | 11 | 8.55 | 94.00 | 6 | 9.83 | 59.00 | 0 |
| TD1 – TD7 | 10 | 9.30 | 93.00 | 7 | 8.57 | 60.00 | 0 |
| TD2 – TD7 | 12 | 7.33 | 88.00 | 5 | 13.00 | 65.00 | 0 |

**Supplementary Table S1 Panel C note:** N = 17 for all comparisons. Pairwise comparisons were performed using two-sided Wilcoxon signed-rank tests (α = 0.05). Positive ranks indicate participants for whom the first listed session had a higher mean IES than the second. Negative ranks indicate participants for whom the first listed session had a lower mean IES than the second. Ties indicate equal values. Mean ranks and sums of ranks are based on non-zero paired differences. IES = inverse efficiency score.

**Supplementary Table S2: Model 1 Baseline TEP amplitude and orthogonalized acute TEP change predicting Day 1 IES learning slope**

| **TEP component** | **Predictor** | **B** | **β** | **t** | **Raw p** | **FDR q** | **Partial r** | **Model R²** | **Model F(2,13)** | **Model p** |
| --- | --- | --- | --- | --- | --- | --- | --- | --- | --- | --- |
| N45 | Baseline amplitude (Pre) | -6.431 | -0.490 | -2.029 | 0.063 | 0.445 | -0.490 | 0.243 | 2.086 | 0.164 |
|  | Orthogonalized dTEP (dTEP⊥) | 1.291 | 0.056 | 0.231 | 0.821 | 0.976 | 0.056 |  |  |  |
| N100 | Baseline amplitude (Pre) | −4.891 | −0.373 | -1.465 | 0.167 | 0.445 | −0.376 | 0.160 | 1.237 | 0.322 |
|  | Orthogonalized dTEP (dTEP⊥) | 3.915 | 0.166 | 0.652 | 0.526 | 0.976 | 0.178 |  |  |  |
| P180 | Baseline amplitude (Pre) | 0.094 | 0.009 | 0.031 | 0.976 | 0.976 | 0.009 | 0.001 | 0.006 | 0.994 |
|  | Orthogonalized dTEP (dTEP⊥) | 0.282 | 0.028 | 0.099 | 0.923 | 0.972 | 0.027 |  |  |  |
| N280 | Baseline amplitude (Pre) | 6.436 | 0.403 | 1.588 | 0.136 | 0.445 | 0.403 | 0.162 | 1.261 | 0.316 |
|  | Orthogonalized dTEP (dTEP⊥) | -0.146 | -0.009 | -0.036 | 0.972 | 0.976 | -0.010 |  |  |  |

**Supplementary Table S2 note:** Model 1 consisted of separate multiple linear regression models for each TEP component (n = 16). The dependent variable was the Day 1 IES learning slope across training blocks 1–12. Pre represents baseline TEP amplitude measured immediately before Day 1 motor learning. dTEP = Pre − Post, and dTEP⊥ represents acute TEP change orthogonalized with respect to baseline amplitude. B = unstandardized regression coefficient; β = standardized regression coefficient; r = correlation coefficient. Two-tailed p-values are reported, α was set at 0.05.

**Supplementary Table S3: Model fit, covariance estimates, and diagnostics for Model 2**

| **TEP component** | **N participants** | **N interval observations** | **−2LL** | **AIC** | **BIC** | **Residual variance:  24-h interval** | **Residual variance:  5-day interval** | **Convergence** | **Diagnostic assessment** |
| --- | --- | --- | --- | --- | --- | --- | --- | --- | --- |
| N45 | 17 | 32 | 318.354 | 322.354 | 324.870 | 26,562.971 | 1,270.211 | Converged | No convergence warnings |
| N100 | 17 | 32 | 313.822 | 317.822 | 320.338 | 21,959.092 | 1,305.542 | Converged | No convergence warnings |
| P180 | 17 | 32 | 313.562 | 317.562 | 320.079 | 19,242.945 | 1,203.911 | Converged | No convergence warnings |
| N280 | 17 | 32 | 315.296 | 319.296 | 321.812 | 21,896.247 | 1,298.680 | Converged | No convergence warnings |

**Supplementary Table S3 note:** Model 2 was estimated separately for each TEP component using restricted maximum likelihood (REML) with Satterthwaite denominator degrees of freedom. Repeated interval-level observations were modeled using a diagonal covariance structure, allowing separate residual variances for the 24-h (TD1→TD2) and 5-day (TD2→TD7) intervals while constraining the residual covariance between intervals to zero. −2LL, −2 log likelihood; AIC, Akaike information criterion; BIC, Bayesian information criterion. Model-fit indices are provided descriptively and are not intended for comparison across TEP-component models with different predictor values.

**Supplementary Table S4: Component-specific repeated-measures Model 2 estimates for subsequent IES performance**

| **Component** | **Fixed effect** | **B** | **SE** | **df** | **t** | **Raw p** | **95% CI** | **FDR q*** |
| --- | --- | --- | --- | --- | --- | --- | --- | --- |
| N45 | Interval: 5-d vs 24-h | 46.144 | 44.130 | 14.595 | 1.046 | 0.313 | -48.144, 140.433 | — |
|  | Prior IES slope | 0.050 | 0.060 | 13.022 | 0.830 | 0.422 | -0.080, 0.180 | — |
|  | Pre | 6.312 | 27.374 | 13.139 | 0.231 | 0.821 | -52.762, 65.385 | 0.821 |
|  | dTEP (24-h slope) | 89.320 | 209.699 | 13.283 | 0.426 | 0.677† | -362.730, 541.369 | 0.751* |
|  | Interval × dTEP | −71.441 | 210.137 | 13.393 | -0.340 | 0.739 | -524.062, 381.180 | 0.806 |
| N100 | Interval: 5 d vs 24 h | 75.161 | 41.237 | 14.589 | 1.823 | 0.089 | -12.951, 163.272 | — |
|  | Prior IES slope | 0.046 | 0.065 | 13.144 | 0.698 | 0.497 | -0.095, 0.186 | — |
|  | Pre | 39.440 | 57.550 | 14.252 | 0.685 | 0.504 | -83.789, 162.668 | 0.751 |
|  | dTEP (24-h slope) | 478.686 | 265.030 | 13.814 | 1.806 | 0.093† | -90.467; 1047.838 | 0.204* |
|  | Interval × dTEP | −463.289 | 264.102 | 13.648 | -1.754 | 0.102 | -1031.104, 104.526 | 0.204 |
| P180 | Interval: 5 d vs 24 h | 14.532 | 39.787 | 14.397 | 0.365 | 0.720 | -70.581, 99.645 | — |
|  | Prior IES slope | 0.093 | 0.074 | 12.966 | 1.265 | 0.228 | -0.066, 0.253 | — |
|  | Pre | 36.411 | 43.504 | 13.888 | 0.837 | 0.417 | -56.967, 129.788 | 0.715 |
|  | dTEP (24-h slope) | −281.069 | 110.920 | 13.810 | −2.534 | 0.024† | −519.276, −42.862 | 0.204* |
|  | Interval × dTEP | 253.116 | 110.710 | 13.752 | 2.286 | 0.039 | 15.266, 490.966 | 0.204 |
| N280 | Interval: 5 d vs 24 h | 65.496 | 39.925 | 14.446 | 1.640 | 0.122 | -19.888, 150.880 | — |
|  | Prior IES slope | 0.058 | 0.062 | 12.992 | 0.942 | 0.363 | -0.075, 0.191 | — |
|  | Pre | −16.478 | 33.022 | 13.412 | -0.499 | 0.626 | -87.595, 54.640 | 0.751 |
|  | dTEP (24-h slope) | 399.623 | 220.605 | 13.019 | 1.811 | 0.093† | -76.895, 876.142 | 0.204* |
|  | Interval × dTEP | −398.042 | 221.877 | 13.320 | -1.794 | 0.096 | -876.208, 80.124 | 0.204 |

**Supplementary Table S4 note:** B = unstandardized coefficient; SE = standard error; CI = confidence interval; Pre = interval-specific pre-task TEP amplitude; dTEP = Pre − Post. The 24-h TD1→TD2 interval was the reference category; therefore, the dTEP coefficient represents the dTEP simple slope during the 24-h interval. Models used a diagonal repeated covariance structure and Satterthwaite denominator degrees of freedom. *FDR q-values were calculated from the corresponding Type III fixed-effect p-values for the 12 prespecified TEP-related tests (Pre, dTEP, and Interval × dTEP across four components); Interval and prior IES slope were excluded from the FDR family. †The displayed raw p-value for dTEP is the coefficient/simple-slope p-value and may therefore differ slightly from the Type III p-value used for FDR correction.
